# Childhood type 1 diabetes mellitus and gut microbiota: from microbiome characteristics to prevention and treatment strategies

**DOI:** 10.3389/fcimb.2026.1873700

**Published:** 2026-07-28

**Authors:** Chaoyan Yang

**Affiliations:** Department of Paediatrics, Luzhou People’s Hospital, Luzhou, China

**Keywords:** children, gut microbiota, immune regulation, probiotics, type 1 diabetes mellitus

## Abstract

Type 1 diabetes mellitus (T1DM) in children is a chronic metabolic disease mediated by autoimmune abnormalities, characterized by extensive destruction and impairment of pancreatic islet β-cells, and represents a common chronic endocrine disorder in childhood. The occurrence and progression of T1DM are closely associated with genetic susceptibility and environmental factors. Compared with healthy children, children with T1DM exhibit a disrupted gut microecological balance, characterized by a marked reduction in microbial diversity, structural disturbances in dominant microbiota, and concomitant abnormalities in multiple metabolic pathways. Recent studies have indicated that the gut microbiota, as a key environmental factor, is associated with the pathophysiological processes of pediatric T1DM through mechanisms including altered intestinal permeability and modulation of immune homeostasis. This article provides a comprehensive narrative review of the characteristics of the gut microbiota in children with T1DM, summarizes current evidence regarding microbiome alterations and their underlying mechanisms, and discusses emerging microbiota-targeted interventions. The aim is to provide new insights for the early prevention, control, and clinical management of T1DM in children.

## 1 Introduction

T1DM is a chronic metabolic disorder characterized by autoimmune-mediated destruction of pancreatic β-cells, leading to insulin deficiency, and typically onset during childhood or adolescence (Jacobsen and Schatz, 2026; Veijola et al., 2026). In recent years, the global incidence of T1DM in children has continued to rise, making it a major public health issue that seriously threatens children’s health (Deutsch et al., 2026). Although insulin replacement therapy has significantly improved patient survival, it remains unable to halt disease progression or prevent the development of multisystem complications. It is currently believed that the development of T1DM is driven by a combination of genetic susceptibility and environmental factors (Kim et al., 2026; Vollenbrock et al., 2026). Among these, environmental factors play a significant role in disease progression. As the largest microecosystem in the human body, the gut microbiota plays an irreplaceable role in early-life immune regulation, maintenance of intestinal mucosal barrier function, and metabolic regulation (Nikola and Iva, 2024; Tan et al., 2024). Serving as an important interface between external factors and the host immune response, the gut microbiota contributes to the development of T1DM by regulating immune homeostasis and metabolic pathways (Huang et al., 2024).

A growing body of research indicates that children with T1DM exhibit significant dysbiosis of the gut microbiota, such as reduced microbial diversity, structural changes, and metabolic abnormalities (Zheng et al., 2024; Li et al., 2025). The gut microbiota may contribute to the progression of T1DM through various mechanisms, including disruption of the intestinal mucosal barrier, increased intestinal permeability, and modulation of the host immune response (Fuhri Snethlage et al., 2024; Kumari et al., 2025; Wang et al., 2025). Additionally, early-life interventions can influence the colonization of the gut microbiota (Yau and Danska, 2024). However, the causal relationship between the gut microbiota and T1DM in children remains unclear, and the specific mechanisms of action require further investigation.

Against this backdrop, this article provides a comprehensive narrative review of the relationship between T1DM in children and the gut microbiota. It focuses on the characteristics of the gut microbiota in pediatric T1DM and its potential underlying mechanisms, analyzes environmental factors influencing microbiota changes, and summarizes research progress in microecological modulation, including the use of probiotics. The aim is to provide a scientific basis and clinical insights for the early risk identification and microbiome-targeted intervention in pediatric T1DM.

## 2 Literature search strategy

This narrative review was based on a comprehensive literature search conducted in PubMed, Web of Science, Embase, and Scopus up to January 2026. Search terms included combinations of “type 1 diabetes”, “children”, “pediatric”, “gut microbiota”, “intestinal microbiome”, “dysbiosis”, “probiotics”, “prebiotics”, and “fecal microbiota transplantation”. Priority was given to studies involving children and adolescents (<18 years of age) with T1DM. Adult studies and studies of type 2 diabetes mellitus were included only when relevant pediatric evidence was limited and when they provided important mechanistic insights. Original research articles, clinical studies, cohort studies, and high-quality reviews published in English were considered.

## 3 Characteristics of the gut microbiota in children with T1DM

### 3.1 Microbial diversity and compositional shifts

Gut microbiota dysbiosis has been consistently associated with pediatric T1DM and is considered a potential environmental contributor to disease development (Deutsch et al., 2026). Regarding microbial diversity, most studies have found that children with T1DM exhibit significantly reduced α-diversity in their gut microbiota, manifested as decreased species richness and evenness (Kunath et al., 2022; Fliegerová et al., 2025). This state of low diversity may impair the microbiota’s ability to modulate the host immune system. For example, a case-control study of newly diagnosed children with T1DM in China showed that fecal bacterial richness (Chao index) in the T1DM group was significantly lower than in the control group (Qi et al., 2016). Concurrently, β-diversity analysis indicates a distinct separation in community structure between children with T1DM and healthy controls (Lakshmanan et al., 2021). Although longitudinal cohorts such as TEDDY and DIPP suggest that microbiota alterations may precede islet autoimmunity, these findings remain observational and do not establish causality (Stewart et al., 2018).

In terms of microbial composition, children with T1DM generally exhibit an imbalance characterized by a reduction in beneficial bacteria and an increase in opportunistic pathogens (Lv et al., 2020; Gradisteanu Pircalabioru et al., 2021). There is a significant decrease in beneficial bacteria that produce short-chain fatty acids, including a reduction in the abundance of anti-inflammatory genera such as *Faecalibacterium* and *Roseburia* within the *Firmicutes* phylum (Tan et al., 2024). Conversely, the abundance of the *Bacteroidetes* phylum and certain opportunistic pathogens increased, leading to a decrease in the *Bacteroidetes*/*Bacteroides* ratio (Leiva-Gea et al., 2018). In children with T1DM, the abundance of *Bacteroides* increased, while beneficial bacteria such as *Bifidobacterium*, *Lactobacillus*, and *Prevotella* decreased (Asante Baadu et al., 2025). A comparative study of children carrying at least two diabetes-associated autoantibodies versus matched autoantibody-negative controls found that reduced abundance of lactate-producing species, scarcity of *Bifidobacterium adolescentis* and *Bifidobacterium pseudocatenulatum*, and increased abundance of *Bacteroides* were associated with β-cell autoimmunity (de Goffau et al., 2013). A Chinese study of children further found that the percentage of the genus *Blautia* was positively correlated with HbA1c levels, the number of autoantibodies, and titers of tyrosine phosphatase autoantibodies (Qi et al., 2016). In contrast, studies using non-obese diabetic mouse models provide stronger experimental evidence supporting a causal role of microbiota alterations in autoimmune diabetes development (Sun et al., 2024). Notably, some studies have not found significant differences in diversity or genus abundance between case and control groups (Endesfelder et al., 2014), suggesting that results may be influenced by factors such as population and diet.

### 3.2 Abnormalities in microbial function and metabolic pathways

Functionally, the gut microbiota associated with T1DM in children exhibits significant metabolic reprogramming, with the most representative change being a decline in the capacity to synthesize short-chain fatty acids (SCFAs) (Cui et al., 2024; Yarmohammadi et al., 2025). SCFAs (such as butyrate and propionate) are not only an important energy source for intestinal epithelial cells but also play a central role in maintaining intestinal barrier integrity and regulating immune tolerance (Mukhopadhya and Louis, 2025). Butyrate, in particular, promotes the differentiation of regulatory T cells and suppresses inflammatory responses (Ghorbanian et al., 2025). However, the number of butyrate-producing bacteria in the intestines of children with T1DM is significantly reduced, leading to decreased SCFA levels. This, in turn, weakens intestinal barrier function and increases permeability, facilitating the trans-barrier transport of antigens and activating autoimmune responses. Concurrently, reduced SCFAs can also affect free fatty acid receptor signaling pathways, decreasing glucagon-like peptide-1 secretion, which further impacts pancreatic β-cell function and glucose metabolism regulation, exacerbating glucose homeostasis disruption (Del Chierico et al., 2022; Yuan et al., 2022).

In addition to abnormal SCFA metabolism, several key metabolic pathways also exhibit systemic dysregulation. Upregulation of the lipopolysaccharide (LPS) biosynthesis pathway is another significant feature (Tian et al., 2001). A microbiota composition rich in Gram-negative bacteria can increase LPS release, inducing metabolic endotoxemia, which triggers chronic low-grade inflammation by activating the Toll-like receptor 4 (TLR4) pathway and promotes autoimmune processes (Singh et al., 2015). Concurrently, amino acid and bile acid metabolic pathways also exhibit significant abnormalities (Yao et al., 2025). Overall, abnormalities in gut microbiota function and metabolic pathways constitute a crucial link between microbiome dysbiosis and host immune dysregulation, playing a key role in the onset and progression of T1DM in children.

In summary, the gut microbiota associated with T1DM in children not only undergoes structural changes but also exhibits systemic abnormalities at the functional and metabolic levels. These changes suggest a potential link between gut dysbiosis and immune dysfunction in type 1 diabetes. **Table 1** summarizes the composition and functional characteristics of the gut microbiota in children with T1DM.

**Table 1:** Gut microbiota characteristics in children with T1DM.

Author/Date	Study design	Subjects	Age range	Content	Findings	Ref.
Davis-Richardson AG et al/2014	case-control	T1DM (n=76), HC (n=47)	0.4–2.2 y	16S rRNA sequencing analysis of gut microbiota differences between two patient groups before the onset of autoimmunity.	Bacteroides dorei abundance rises significantly before autoantibodies in T1DM children.	(Davis-Richardson et al., 2014)
Murri M et al/2013	case-control	T1DM (n=16), HC (n=16)	Mean 7.2–7.5 y	PCR-DGGE and qPCR analysis of fecal microbiota composition and its correlation with blood glucose levels between two groups.	Altered T1DM microbiota: fewer Bifidobacterium/Lactobacillus (negatively linked to glucose) (*P < 0.05*), more Clostridium.	(Murri et al., 2013)
Yuan X et al/2022	cohort	T1DM (n=64), HC (n=77)	Mean 7.5–7.9 y	Multi-omics analysis+FMT validation.	T1DM children: reduced butyrate/bile acid, increased LPS synthesis (*P < 0.05*); FMT induces mouse glucose dysmetabolism.	(Yuan et al., 2022)
Liu X et al/2021	case-control	T1DM (n=51), HC (n=47)	6–14 y	16S rRNA sequencing analysis of gut microbiota structure and functional changes, and correlation with FBG.	Higher microbiota diversity in T1DM children; specific genera linked to glucose; dysfunction in glycolipid pathways.	(Liu et al., 2021)
Leiva-Gea I et al/2018	case-control	T1DM (n=15), MODY2 (n=15), HC (n=13)	Mean 12.3–13.1 y	16S rRNA sequencing: microbiota composition/function, inflammatory cytokines, LPS, gut permeability among 3 groups.	Lower microbiota diversity in T1DM children: fewer beneficial bacteria, more potential pathogens, with inflammation and gut barrier damage.	(Leiva-Gea et al., 2018)
Harbison JE et al/2019	cohort	Autoimmunity/T1DM (n=47), HC (n=41)	Mean 10.7–11.8 y	Longitudinal analysis: gut microbiota & plasma SCFAs.	T1DM children: reduced microbiota diversity, fewer SCFA-producing bacteria (*P < 0.05*), and increased gut permeability.	(Harbison et al., 2019)
Tamahane V et al/2024	case-control	T1DM (n=68), HC (n=61)	Mean 12.1–12.4 y	16S rRNA sequencing compares microbiota composition between two groups and analyzes genus abundance in relation to glycemic control.	No diversity difference between T1DM and controls; more Parasutterella; increased Haemophilus linked to lower HbA1c (*P < 0.05*).	(Tamahane et al., 2024)
Traversi D et al/2020	case-control	T1DM (n=40), HC (n=56)	5–10 y	Molecular methods detect fecal microbiota.	T1DM linked to increased Firmicutes and decreased Bifidobacterium.	(Traversi et al., 2020)
Arhire AI et al/2025	case-control	T1DM (n=31)	1–18 y	Assess gut microbiota associations with T1DM, blood glucose, DKA, thyroid autoimmunity, and inflammation.	Dysbiosis in T1DM children: Butyrivibrio and Bacteroides correlate with onset age and progression markers (*P < 0.05*).	(Arhire et al., 2025)

FBG, fasting blood glucose; SCFA, short-chain fatty acids; FMT, fecal microbiota transplantation; LPS, lipopolysaccharide; DKA, diabetic ketoacidosis.

## 4 Potential mechanistic links between the gut microbiota and T1DM in children

### 4.1 Intestinal barrier dysfunction

The intestinal mucosal barrier serves as the body’s first line of defense against exogenous antigens and microbial invasion. It is composed of intestinal epithelial cells, tight junction proteins, the mucus layer, and gut-associated lymphoid tissue (Lan et al., 2023; Arumugam et al., 2025). Under normal physiological conditions, the gut microbiota ensures the integrity of the intestinal barrier by promoting the expression of tight junction proteins and maintaining the thickness of the mucus layer (Nwako et al., 2025). However, during the development of T1DM in children, dysbiosis of the gut microbiota may be associated with disruption of this barrier structure through multiple pathways, leading to increased intestinal permeability (Mønsted et al., 2021; Stojanović et al., 2021). Studies have shown that key bacterial populations producing SCFAs are significantly reduced in children with T1DM, resulting in insufficient levels of metabolites such as butyrate (Tillett et al., 2025). Recent reviews have further emphasized that SCFAs are critical regulators of intestinal barrier maturation and immune homeostasis during childhood, and disturbances in SCFA metabolism may contribute to the development of immune-mediated diseases, including T1DM (Del Chierico et al., 2022; Mukhopadhya and Louis, 2025). As the primary energy source for colonic epithelial cells, a deficiency in butyrate not only impairs the proliferation and repair capacity of epithelial cells but also downregulates the expression of tight junction proteins such as occludin, claudin, and ZO-1 (Hu et al., 2023). This further disrupts intercellular junctional structures and compromises barrier integrity. Additionally, SCFAs play a role in modulating inflammatory responses and maintaining immune tolerance. Their reduction further exacerbates the local inflammatory microenvironment, creating a vicious cycle of barrier damage and inflammatory responses (Lassenius et al., 2017; Moon et al., 2021).

In addition, dysbiosis can also affect the structure and function of the intestinal mucus layer. Under normal conditions, the gut microbiota maintains the thickness of the mucus layer by promoting the secretion of mucins (Luis and Hansson, 2023). However, in the context of T1DM-associated dysbiosis, certain mucus-degrading bacteria increase in relative abundance, leading to a thinning or even rupture of the mucus layer, which exposes intestinal epithelial cells directly to microorganisms and their metabolites (Gudi et al., 2026). Studies of intestinal organoids derived from children with T1DM have shown that the duodenal epithelium exhibits abnormalities in cell proliferation and differentiation, imbalances in ion transport, and impaired barrier integrity (Bharadiya et al., 2024). Concurrently, endotoxins such as LPS produced by the gut microbiota can enter the bloodstream through the compromised epithelial barrier, activating antigen-presenting cells such as dendritic cells (Kihl et al., 2019). This induces an innate immune response and promotes adaptive immune activation, ultimately triggering autoimmunity against pancreatic β-cells. Therefore, impaired intestinal mucosal barrier function serves as a critical link between gut microbiota dysbiosis and systemic immune abnormalities.

### 4.2 Immune dysregulation

During childhood, the immune system undergoes a dynamic maturation process. As a key source of immunostimulatory microorganisms, the gut microbiota plays a central role in establishing immune tolerance and defensive capabilities (Rooks and Garrett, 2016; Pagliari et al., 2018). Under normal conditions, the diverse microbiota that colonizes early in life promotes the appropriate maturation of the intestinal mucosal immune system and suppresses excessive inflammatory responses through the regulatory activity of secretory IgA (Huang et al., 2020). However, children with T1DM often exhibit a reduction in SCFA-producing bacteria, leading to impaired regulatory T cell (Treg) differentiation and difficulty in establishing immune tolerance. A study of jejunal biopsies from 31 children with type 1 diabetes revealed that, even in intestinal mucosa with normal structure and no celiac disease, the expression of immune molecules such as HLA-DR, HLA-DP, ICAM-1, IL-1α, and IL-4 was elevated, while IL-2 and IFN-γ were associated with the severity of celiac disease (Westerholm-Ormio et al., 2003).

Concurrently, impaired microbial barrier function allows substances such as LPS to enter the circulation, persistently activating the TLR4/NF-κB pathway and inducing the release of pro-inflammatory cytokines such as IL-1β, IL-6, and TNF-α (Gomes et al., 2018). The NLRP3 inflammasome is also abnormally activated, further exacerbating chronic low-grade inflammation (Ye et al., 2022). These findings support the role of the intestinal immune system in the pathogenesis of type 1 diabetes. At the adaptive immune level, an imbalance in the Treg/Th17 ratio leads to the expansion of pro-inflammatory Th17 cells and increased levels of effector molecules such as IL-17, promoting local inflammatory infiltration of the islets (Starosz et al., 2022). These immune dysregulations collectively mediate islet inflammatory infiltration and β-cell destruction, while simultaneously exacerbating local intestinal inflammation, driving the onset of diabetes, and forming a pathological gut-immune-islet cycle (Vaarala et al., 2008).

### 4.3 Microbial metabolites as signaling mediator

The gut microbiota may serve as a signaling mediator in host immune regulation through its metabolic products, serving as a vital link between microbial structure and functional effects (Zuo et al., 2025). Emerging evidence suggests that microbial metabolites are particularly important during childhood, when gut microbiota maturation and immune development occur simultaneously, thereby exerting long-term effects on metabolic and autoimmune diseases (Del Chierico et al., 2022). SCFAs, such as butyrate, propionate, and acetate, are among the most studied metabolic products. Butyrate promotes Foxp3 expression by inhibiting histone deacetylases, enhances the differentiation of regulatory T cells (Tregs), and upregulates the expression of tight junction proteins (such as claudin-1 and occludin), thereby maintaining the integrity of the intestinal epithelial barrier (Campbell et al., 2020). In children with T1DM, there is a reduction in SCFA-producing bacteria, leading to decreased SCFA levels, which in turn causes intestinal barrier damage and immune dysregulation (Abuqwider et al., 2025).

Furthermore, as a component of the Gram-negative bacterial cell wall, elevated levels of LPS serve as a key indicator of dysbiosis and can induce local immune cell infiltration in the islets via the TLR4-NF-κB pathway, thereby accelerating β-cell damage (Chen et al., 2017). Indole compounds produced by tryptophan metabolism regulate intestinal immune homeostasis by activating the aryl hydrocarbon receptor (AhR) (Agus et al., 2018). In T1DM, this pathway is impaired, further exacerbating immune dysregulation. Metabolites such as secondary bile acids and branched-chain amino acids may also contribute to disease progression through their interaction with specific receptors or by reprogramming immune cell metabolism (Liu et al., 2025). Microbial metabolites collectively promote the development of T1DM in children by modulating the intestinal barrier and immune responses.

**Figure 1:**
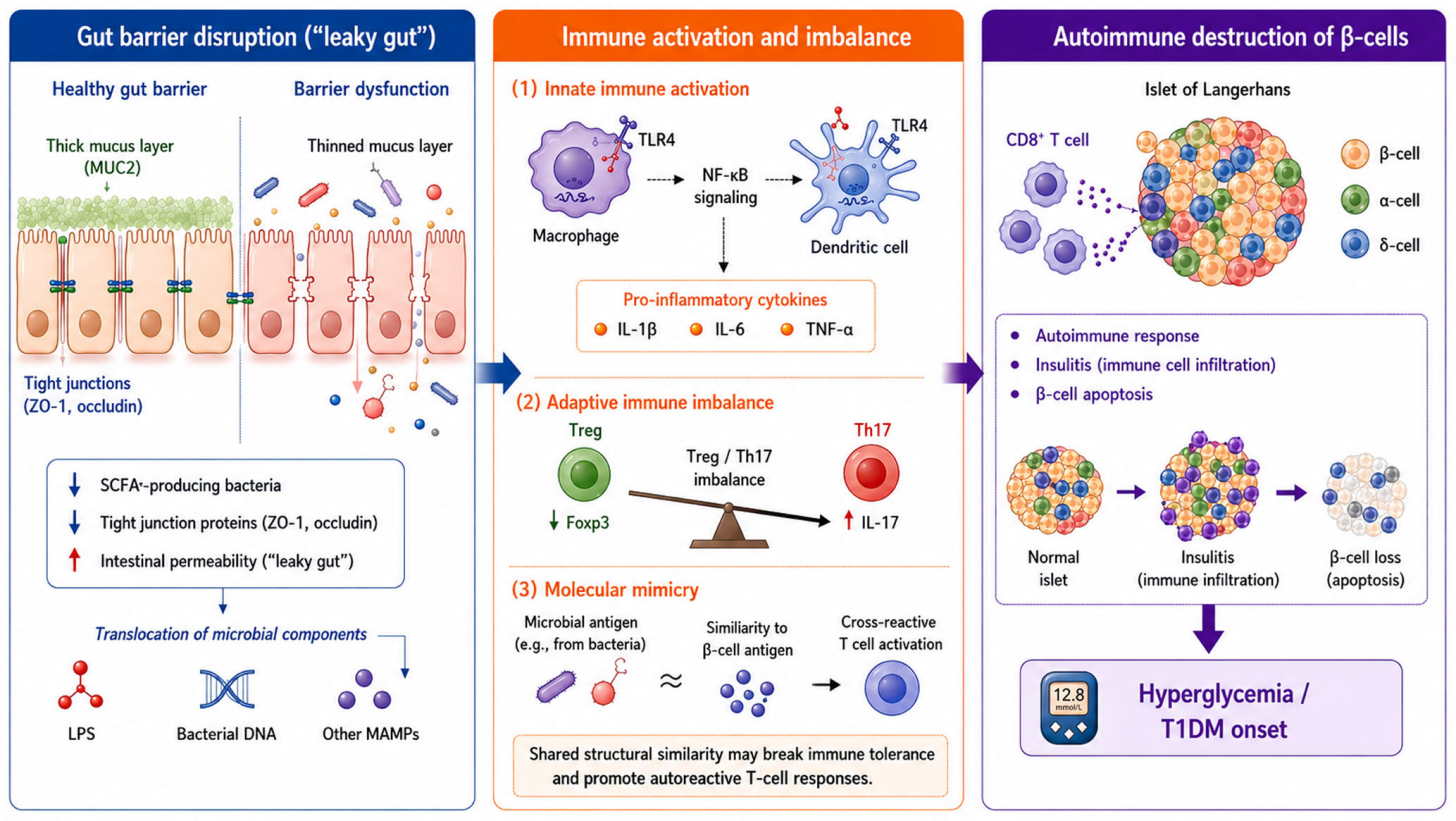
Schematic diagram illustrating the mechanism by which gut microbiota dysbiosis contributes to the development of type 1 diabetes in children. SCFAs, short-chain fatty acids; LPS, lipopolysaccharide; MAMPs, microbe-associated molecular patterns; TLR4, Toll-like receptor 4; NF-κB, nuclear factor kappa B; Treg, regulatory T cells; Th17, T helper 17 cells.

In summary, the gut microbiota disrupts the intestinal mucosal barrier, induces an imbalance in immune regulation, and alters metabolic products, thereby establishing a network of interactions that collectively contribute to the development of T1DM in children. **Figure 1** illustrates the process by which gut dysbiosis promotes the development of T1DM in children through these multiple mechanisms.

## 5 Microbiome-targeted interventions

### 5.1 Probiotics and prebiotics

Probiotics and prebiotics are currently the most widely used strategies for microbiome intervention and have shown promise in the prevention and treatment of T1DM in children (Vitti-Ruela et al., 2023). As active microorganisms beneficial to the host, probiotics can exert immunomodulatory, antioxidant, and pathogen-inhibiting effects via the gastrointestinal tract (Ljungberg et al., 2006). In the context of T1DM, probiotics improve insulin sensitivity and metabolic status by increasing the abundance of beneficial bacteria such as *Lactobacillus* and *Bifidobacterium*, correcting microbial dysbiosis, reducing pro-inflammatory cytokine levels, and promoting the secretion of SCFAs and glucagon-like peptide-1 (Bordalo Tonucci et al., 2017). SCFAs play a central role in this process, not only providing energy to intestinal epithelial cells but also enhancing the expression of tight junction proteins and repairing the intestinal mucosal barrier. Animal studies have shown that specific probiotic formulations (such as VSL#3 or *Lactobacillus casei*) can reduce the incidence of diabetes in non-obese mice and protect β-cells (Calcinaro et al., 2005). Population-based studies also suggest that early postnatal probiotic supplementation is associated with a reduced risk of pancreatic islet autoimmunity (Uusitalo et al., 2016).

Prebiotics are dietary components (such as fructooligosaccharides and inulin) that are not digested by the host but can be selectively utilized by beneficial gut bacteria (Snelson et al., 2021). They indirectly regulate the gut microbiota by promoting the proliferation of endogenous beneficial bacteria (Megur et al., 2022). The SCFAs produced through their fermentation help maintain the integrity of the intestinal barrier and regulate the secretion of intestinal hormones, thereby improving insulin sensitivity and blood glucose control (Liu et al., 2017). The combination of probiotics and prebiotics, known as synbiotics, can produce synergistic effects.

Randomized controlled trials suggest that specific probiotic or synbiotic interventions may improve microbial diversity and reduce inflammatory markers, and some studies reported modest protective effects on C-peptide levels, although these findings are not consistent across all trials (Groele et al., 2021; Nikola and Iva, 2024). Prebiotics alone have shown limited effects on glycemic control and intestinal permeability, with mean HbA1c reductions typically ranging from approximately 0.1% to 0.5% in small pediatric trials (Ho et al., 2019; Kumar et al., 2021). The effects vary substantially depending on the probiotic strains, combinations, and dosing regimens. For example, supplementation with *Lactobacillus rhamnosus GG* or *Bifidobacterium lactis Bb12* did not demonstrate significant improvements in β-cell function in newly diagnosed children (Mondanelli et al., 2020; Groele et al., 2021), whereas multi-strain synbiotic formulations combining *Lactobacillus*, *Bifidobacterium*, and prebiotic fibers showed modest reductions in HbA1c and inflammatory markers in some studies (Zare Javid et al., 2020; Adly et al., 2025).

Overall, while microbiome-based interventions hold potential for modulating gut microbiota and metabolic outcomes in children with T1DM, current evidence does not support their routine clinical use. More standardized, multicenter, randomized controlled trials are needed to clarify their efficacy, safety, optimal strains and dosing, and suitable patient populations. **Table 2** summarizes clinical and animal studies on the effects of probiotic/prebiotic interventions on the gut microbiota.

**Table 2:** Effects of probiotic/prebiotic intervention on the gut microbiota.

Probiotic/Prebiotic	Study design	Subjects	Intervention/Aim	Findings	Ref.
Lactobacillus salivarius subsp. salicinius AP-32, L. johnsonii MH-68, Bifidobacterium subsp. lactis CP-9	RCT	56 T1DM patients (6–18 y)	Evaluate adjuvant effects on glucose, inflammatory cytokines, and microbiota.	Reduced glucose, HbA1c, inflammatory cytokines (*P < 0.05*); increased beneficial bacteria.	(Wang et al., 2022)
Oligofructose-enriched inulin	RCT	43 T1DM patients (8–17 y)	Evaluate effects on HbA1c, C-peptide, gut microbiota, permeability, hypoglycemia frequency.	Increased C-peptide (*P < 0.05*), slight gut permeability improvement, more *Bifidobacterium*; no clear glycemic control improvement	(Ho et al., 2019)
Lactobacillus acidophilus	RCT	70 T1DM children (5–18 y)	Assess effects on FBG, HbA1c, lipids, IL-21, IL-22.	Decreased FBG, HbA1c, total cholesterol, IL-21; increased IL-22 (*P < 0.05*).	(Adly et al., 2025)
lactobacillus rhamnosus GG	RCT	61 T1DM children (3–18 y)	Assess effects on serum tryptophan and inflammatory cytokines.	Altered tryptophan metabolism, reduced inflammatory cytokine production (*P < 0.05*).	(Mondanelli et al., 2020)
Lactobacillus sporogenes GBI-30, maltodextrin, and fructooligosaccharide	RCT	50 T1DM patients (4–18 y)	Assess effects on glucose, HbA1c, lipids, oxidative stress markers.	Improved blood glucose and oxidative stress; reduced inflammatory markers	(Zare Javid et al., 2020)
Visbiome®	RCT	96 new-onset T1DM children (2–12 y)	Evaluate multiple probiotic strains on HbA1c, C-peptide, insulin dose.	Improved blood glucose, reduced insulin requirement (*P < 0.05*).	(Kumar et al., 2021)
Vivomixx®	RCT	60 new-onset T1DM children (2–12 y)	Assess effects on iTregs%, autoantibodies, IL-10, C-peptide, HbA1c.	Increased iTregs and IL-10 (*P < 0.05*), improved β-cell function.	(Lokesh et al., 2025)
Bifidobacterium spp.	animal model	STZ-induced diabetic C57BL/6J mice	Evaluate effects on glucose, MAPK pathway, adipokines.	Lowered blood glucose (*P < 0.05*), activated insulin signaling, anti-inflammatory effects.	(Le et al., 2015)
Human milk oligosaccharides	animal model	Non-obese diabetic (NOD) mice	Assess effects on T1DM development, fecal microbiota, SCFAs, cytokines.	Delayed T1DM onset via modulation of microbiota, SCFAs, and immune cells.	(Xiao et al., 2018)

Effects of probiotics/prebiotics vary substantially across studies. Some trials reported no improvement in β-cell function or C-peptide levels. Heterogeneity is observed in strains, combinations, dosing, and outcomes. RCT, Randomized Controlled Trial.

### 5.2 Dietary modulation

Diet is a key environmental factor in shaping the structure and function of the gut microbiota, and diet-based microbiome interventions hold significant potential for the prevention and treatment of T1DM in children (Zhao et al., 2018). Dietary interventions can partially restore gut microbiota symbiosis and related metabolic functions, and alleviate metabolic inflammation. A high-fiber diet provides substrates for fermentation, significantly promotes the production of short-chain fatty acids, enhances intestinal barrier integrity, and inhibits the growth of opportunistic pathogens (Chen et al., 2023). Diets rich in plant-based fiber and complex carbohydrates favor the formation of a *Prevotella*-dominant microbiota, whereas Western diets rich in animal protein and saturated fats promote a *Bacteroides*-dominant microbiota, which is associated with an increased risk of T1DM (Chu et al., 2025). Breastfeeding plays a decisive role in shaping the gut microbiota during early life. Human milk oligosaccharides, which are abundant in breast milk, selectively promote the proliferation of beneficial bacteria such as *Bifidobacterium*, thereby facilitating immune maturation (Flaherman et al., 2018). Epidemiological studies suggest that breastfeeding is associated with a reduced risk of developing T1DM (Moorhead et al., 2024). Overall, the effectiveness of dietary interventions is influenced by baseline gut microbiota composition and individual adherence. Further long-term intervention studies targeting children with T1DM are needed to establish the optimal dietary regimen.

### 5.3 Fecal microbiota transplantation

Fecal microbiota transplantation (FMT) may helps increase beneficial metabolites such as short-chain fatty acids, reduce LPS levels, and suppress inflammatory responses, thereby potentially delaying autoimmune-mediated damage to pancreatic β-cells (Høyer et al., 2025). Existing studies, primarily involving adults and older adolescents with newly diagnosed T1DM, suggest that FMT may delay the decline in endogenous insulin secretion within 12 months of diagnosis and help preserve residual β-cell function (de Groot et al., 2021). However, these findings are based on a limited number of studies with relatively small sample sizes and short follow-up periods. In addition, current clinical evidence remains limited, and studies in pediatric populations are predominantly exploratory in nature. Therefore, FMT should currently be regarded as a promising but still investigational therapeutic approach. At present, FMT still faces challenges such as donor selection, lack of protocol standardization, potential infection risks, and uncertainties regarding its long-term efficacy and safety.

## 6 Prospects and limitations

Although existing studies suggest that the gut microbiota alterations are associated the onset and progression of T1DM in children, current evidence remains significantly limited. First, most available studies are cross-sectional or small-sample observational studies, making it difficult to establish a causal relationship between microbiota dysbiosis and the disease development. Second, substantial heterogeneity exists among studies due to differences in geographic regions, age groups,

dietary habits, antibiotic exposure, medication use (including insulin regimens), comorbid conditions, and genetic backgrounds, which may confound observed associations. In addition, methodological and technical variability related to sample collection, targeted 16S rRNA regions, sequencing platforms, bioinformatic pipelines, and data processing methods may contribute to inconsistent findings across studies. The large number of microbial taxa and metabolites analyzed simultaneously also raises concerns regarding multiple-testing bias and false-positive results. Furthermore, the specific bacterial strains, metabolites, and signaling pathways through which the gut microbiota may influence immune regulation remain incompletely understood. Future research should involve large-scale, multicenter, prospective cohort studies that integrate metagenomics, metabolomics, and transcriptomics to systematically elucidate the mechanisms underlying the interactions between the gut microbiota and the host. Concurrently, further experimental studies are needed to clarify the efficacy and safety of microbiome-based intervention strategies in the prevention and treatment of T1DM. Ultimately, this will lead to new breakthroughs in the precision management of T1DM in children.

## 7 Conclusion

Children with T1DM exhibit dysbiosis and functional impairments in their gut microbiota, which may be associated with the onset and progression of the disease through mechanisms such as disruption of the intestinal barrier and induction of immune dysregulation. Targeting the gut microbiota and its metabolites holds promise as a new strategy for the early prevention and intervention of T1DM in children. Current evidence from probiotics, prebiotics, dietary interventions, and fecal microbiota transplantation suggests potential benefits in modulating gut microbial composition, improving metabolic profiles, and regulating immune responses; however, most findings are derived from small-scale studies or animal models. However, high-quality longitudinal studies and randomized controlled trials are still needed to validate its clinical translational value.
